# Are health and demographic surveillance system estimates sufficiently generalisable?

**DOI:** 10.1080/16549716.2017.1356621

**Published:** 2017-08-18

**Authors:** Philippe Bocquier, Osman Sankoh, Peter Byass

**Affiliations:** ^a^ Centre de recherche en démographie, Université catholique de Louvain, Louvain-la-Neuve, Belgium; ^b^ School of Public Health, Faculty of Health Sciences, University of the Witwatersrand, Johannesburg, South Africa; ^c^ Secretariat, INDEPTH Network, Accra, Ghana; ^d^ Department of Mathematics and Statistics, Njala University, Njala, Sierra Leone; ^e^ Umeå Centre for Global Health Research, Epidemiology and Global Health, Umeå University, Umeå, Sweden

**Keywords:** Generalisation, HDSS, longitudinal data, causal inference

## Abstract

Sampling rules do not apply in a Health and Demographic Surveillance System (HDSS) that covers exhaustively a district-level population and is not meant to be representative of a national population. We highlight the advantages of HDSS data for causal analysis and identify in the literature the principles of conditional generalisation that best apply to HDSS. A probabilistic view on HDSS data is still justified by the need to model complex causal inference. Accounting for contextual knowledge, reducing omitted-variable bias, detailing order of events, and high statistical power brings credence to HDSS data. Generalisation of causal mechanisms identified in HDSS data is consolidated through systematic comparison and triangulation with national or international data.

A Health and Demographic Surveillance System (HDSS) is a ‘geographically-defined population under continuous demographic monitoring with timely production of data on all births, deaths and migrations’ [1] Because HDSS sites are not samples, extrapolating indicators to national level is difficult. However, extrapolating causal mechanisms is possible.

In representative survey data, the sampled population is drawn from a ‘universe’, which is usually the national population. Each unit in this population is interchangeable and a random draw ensures that the sampled units taken as a whole represent the universe well. Confidence intervals account for both sampling errors (associated with sample size, stratification, clustering, etc.) and data collection errors (associated with respondents, interviewers, data entry clerks, etc.) as long as they are random, i.e. non-biased [2].

These sampling rules do not apply in an HDSS framework where the population of a geographically limited area is entirely monitored. The HDSS is considered illustrative of a particular situation monitored through a careful examination of contextual, environmental, and community-level information. HDSSs are usually situated in deprived rural, semi-urban or urban areas. Statistical causal inference in an HDSS is conditional on a well-identified context.

Sampling errors are absent in an HDSS population that is purposively selected and exhaustively followed up. However, there will always remain random data collection errors, expected to be reduced through regular waves of data collection and complex consistency checks. More importantly, randomness occurs from behaviours themselves, which justifies a probabilistic perspective on exhaustive data. A given HDSS population is considered as a unique draw from the universe of all possible situations, also called a ‘super-population’. Therefore, confidence intervals may still be derived through standard error computation techniques (e.g. bootstrap, jack-knife) and will differ from standard sample Gaussian-based estimation [3].

The epistemic framework of conditional causal inference is not only referring to the local nature of HDSS data, but also to the predominant use of regression analysis for statistical inference based on longitudinal data [4]. Rather than aiming at representing the health and demographic status of the national or even sub-national population, HDSSs aim at approaching causal relationships by examining sequences of events in great detail, particularly rare events (e.g. maternal deaths, neglected or emerging diseases), in a population subjected to the same local context. Restricting observations to a geographically limited area and homogenous context avoids omitted or unobservable variables affecting measurements of phenomena of interest. Additionally, contextual variables (e.g. environmental, social) are more easily and efficiently collected locally than nationally. The need for ‘control’ explains why HDSSs have been instrumental for testing public health interventions and conducting phase-IV trials in real populations. An HDSS can monitor incidence (trends) better than prevalence (levels) of health indicators (as done in national sample surveys). Causal relationships between events at community, household, and individual levels are of greater interest in an HDSS than precise descriptions of events at given times. Thus, HDSS analyses aim at causal inference rather than establishing time-specific health and social status.

Another technical issue that HDSSs help to solve is that of statistical power. Statistical power is defined as the inverse of the false negative probability (showing no difference even though it exists). Lack of statistical power is often neglected in statistical analysis in deference to significance or p-values, which is false positive probability (showing a difference where there is none). Many longitudinal sample datasets do not provide minimum statistical power of 80% [5]. Exceptions are the demographic and health surveys (DHS) in which female samples are equivalent to the number of females followed up in HDSS (median size around 12 000 in the 2000s). However, longitudinal analysis in HDSS is not limited to fertility or child mortality. For example, overall mortality or migration analyses show statistical power close to 100%, bringing minimal risk of false negatives and giving greater confidence in identified significant differences.

Despite the above, causal relationships inferred from HDSS data may be too site-specific even for conditional generalisation. Idiosyncrasies inherent in each HDSS can be compensated by comparative analyses and cross-validations of HDSS data, as facilitated by the INDEPTH Network [6]. Standardised data collection and data analysis procedures help here [7]. Cross-site comparisons give remarkable insights on behavioural variations and stability [8]. Triangulation with national administrative, hospital, census and survey data may also help generalise HDSS results [9]. Comparison of INDEPTH cause-of-death data with Global Burden of Disease estimates (national levels derived from modelling heterogeneous data) showed extensive congruence [10]. Analyses of contextual variables (e.g. distance to cities, GDP per capita, population density and other environmental conditions) in 39 HDSSs showed that HDSSs were quite representative across sub-Saharan Africa despite non-random locations [11].

In conclusion, the national, population-based approach of representativeness should not dominate the debate about the usefulness of HDSS data. Accounting for contextual knowledge, reducing omitted-variable bias, detailing order of events and high statistical power brings credence to these data, provided that generalisation is consolidated through systematic comparison and triangulation with national or international data.

## Paper context

District-level HDSS data have been challenged as not representing national populations. This paper argues that conditional generalisation best applies to HDSS data, which satisfy the need for high covariate control in causal inference. Comparison and triangulation with other data sources remain useful tools to consolidate HDSS findings.
